# Private Dental Practitioners’ Experience in a Dental Practice-Based Research Network: A Qualitative Evaluation

**DOI:** 10.3390/healthcare14080979

**Published:** 2026-04-08

**Authors:** Valérie Szönyi, Brigitte Grosgogeat, Franck Decup, Jean-Noël Vergnes, Anne-Margaux Collignon

**Affiliations:** 1 Faculté d’Odontologie, University Claude Bernard Lyon 1, 69100 Lyon, France; 2 Service de Soins et de Consultations Dentaires, Hospices Civils de Lyon, 69373 Lyon, France; 3 Centre d’Epidémiologie et de Recherche en Santé des POPulations, Unité Mixte de Recherche 1295 (Team BIOETHICS), Institut National de la Santé et de la Recherche Médicale, University P. Sabatier, 31000 Toulouse, France; 4 ReCOL, French Dental Practice-Based Research Network, 75017 Paris, France; 5 Laboratoire des Multimatériaux et Interfaces, Unité Mixte Recherche, Centre National Recherche Scientifique 5615, University Claude Bernard Lyon 1, 69100 Lyon, France; 6 Faculté d’Odontologie, Université Paris Cité, 92120 Montrouge, France; 7 Service de Médecine Bucco-Dentaire, Assistance Publique des Hôpitaux de Paris—APHP, 75012 Paris, France; 8 Laboratoire Unité Mixte Recherche 1333 Santé Orale, Inserm, Université Paris Cité, 92120 Montrouge, France; 9 UFR Santé de Toulouse, 31000 Toulouse, France; 10 Service d’Odontologie, Centre Hospitalo-Universitaire de Toulouse, 31300 Toulouse, France; 11 Faculty of Dental Medicine and Oral Health Sciences, McGill University, Montreal, QC H3A 1G1, Canada

**Keywords:** practice-based research network, qualitative research, dentists, private practice, motivation, professional role

## Abstract

**Background/Objectives**: Dental Practice-Based Research Networks (DPBRNs) bridge the gap between academic research and private dental practice, addressing questions relevant to everyday medical care. Despite their growing scientific output, little research has explored the experiences of practitioners engaged in these networks. Our study therefore aims to investigate these practitioners’ perspectives in order to identify strategies for improving investigator recruitment, training and data quality in future DPBRN studies. **Methods**: The qualitative methodology was chosen, and our study adhered to the Standards for Reporting Qualitative Research (SRQR) guidelines. Semi-structured interviews were conducted with dentists who had participated in a DPBRNs study and transcribed before being thematically analysed using Braun and Clarke’s framework. MaxQDA 2022 software was used to facilitate coding of the verbatim quotes. **Results**: Three major themes emerged: (1) obstacles to participation, including time constraints, difficulties in patient recruitment, and a perceived disconnect between academia and private practice; (2) facilitators of engagement, such as strong leadership, logistical support, and a collaborative research environment; and (3) personal benefits, such as skill development, breaking professional routines, and counteracting stereotypes about private practitioners’ involvement in research. **Conclusions**: The findings align with existing literature on medical Practice-Based Research Networks (PBRNs), highlighting logistical and motivational barriers while also emphasizing the importance of social and professional benefits. Notably, although financial compensation or credits for continuing professional development are frequently cited as motivators for research participation, these were not significant concerns for our participants. This study sheds light on the experiences of health practitioners in PBRNs, offering recommendations to overcome challenges through strategies such as accessible training, practical incentives and collaboration opportunities.

## 1. Introduction

Scientific evidence serves as the foundation of clinical practice. Systematic research efforts have established protocols that form the best evidence base, driving the evolution of clinical practice by integrating them into education, continuing professional development and clinical guidelines. In medicine, practice-based research networks (PBRNs) emerged in the 1970s as an essential tool for bridging the gap between academic research and real-world clinical practice [1]. These networks enable collaboration between practicing clinicians and researchers to address questions relevant to everyday medical care. Physicians participating in PBRNs generally report high satisfaction due to the opportunity to enhance patient care, engage in professional growth, and foster a sense of community [2,3]. Factors that facilitate participation include minimal disruption to workflow, access to training, and the ability to apply findings directly to clinical practice [4,5]. However, challenges such as time constraints, high workloads, and concerns about data privacy can hinder participation [6,7].

Oral health professionals play a crucial role in integrating oral health into primary care by contributing to prevention, early detection, and interdisciplinary collaboration. For example, the iDENTify study [8] demonstrated how dentists can effectively screen patients for undiagnosed type 2 diabetes and prediabetes during routine dental visits, highlighting their potential to contribute to systemic disease management. This is why the need for evidence-based practice in dentistry is equally critical [9]. Although most dental research is conducted in academic or hospital settings, the majority of dental care takes place in private practices. This discrepancy raises concerns about the applicability of traditional research to everyday dental practice. Randomised controlled trials, for example, often exclude patients with comorbidities or complex dental histories, limiting their external validity despite providing high-quality evidence [10].

These challenges have been addressed by the development of dental practice-based research networks (DPBRNs) since the 1990s. A recent global mapping of DPBRNs shows that their activity and related publications have increased significantly over the past two decades [11]. This growth underscores the increasing importance of DPBRNs in dental research.

In the United States, the National Dental Practice-Based Research Network (NDPBRN), established in the 2000s [12], has conducted numerous studies involving diverse dentists, practice settings, and patient demographics [13]. This diversity of research has enabled DPBRNs to generate impactful and practice-relevant findings. For instance, DPBRNs have notably advanced knowledge of the detection and treatment of carious lesions in real-world practice [14,15], contributing to the progress of minimally invasive dentistry. In France, the RECOL DPBRN (Réseau d’Études Cliniques en Odontologie) was founded in 2018. Today, RECOL DPBRN comprises over a thousand members, primarily private practice dentists, and has carried out surveys, usability trials, retrospective analyses, and prospective studies [16,17,18].

Despite their growing scientific output, DPBRNs face operational challenges, including securing funding, coordinating protocols, and ensuring standardised data collection. Keeping dentists motivated and balancing research with private practice responsibilities are also significant hurdles [19,20]. While much is known about physician experiences in medical PBRNs, less research has explored the perspectives of dentists in DPBRNs. Although a pilot study has demonstrated the feasibility and potential of DPBRNs [21], it also highlights challenges such as the need for better communication between researchers and practitioners, involving dentists in formulating research questions, and providing research training to ensure meaningful participation and uptake of results [21]. A deeper understanding of dentists’ experiences is thus needed for improving DPBRN organization and effectiveness.

Our study aims to investigate dental clinicians’ perspectives on research participation by examining the barriers, facilitators and motivations involved. Additionally, we aim to identify strategies for enhancing investigator recruitment, training, and data quality in future DPBRN studies.

## 2. Materials and Methods

### 2.1. Study Design and Ethics

A qualitative methodology is particularly relevant for exploring the nuanced experiences and perceptions of participants [22]—in our case, private dental practitioners—and identifying the criteria influencing their participation in clinical research studies. The qualitative approach enables the identification of underlying motivations, barriers, and facilitators, providing an in-depth view that can inform effective engagement strategies for DPBRNs. Our study design and reporting adhered to the Standards for Reporting Qualitative Research (SRQR) guidelines [23] (Supplementary Data).

The original national RESTO DATA study, from which the practitioner sample was drawn, received approval from an ethics committee: Comité de Protection des Personnes (CPP) Île-de-France III (3660-NI 29 January 2019) (15). All participating practitioners signed informed consent forms before taking part in the study. Interviews were conducted, and to ensure confidentiality, no personal characteristics of the participants were included in the data analysis.

### 2.2. Researcher Characteristics and Reflexivity

To ensure reflexivity, it is important to acknowledge the potential influence of the researchers’ backgrounds and experiences [24]. Both lead investigators (AMC and VS) are dentists with more than 10 years of experience in a hospital-university setting. Additionally, one investigator (AMC) participated as a practitioner-researcher in the clinical study from which the sample for this research was drawn, while the other (VS) serves on the association’s board. Throughout all stages of the research, they (AMC and VS) engaged in continuous self-reflection and discussions with the research team (other co-authors) to ensure that their perspectives and assumptions were critically examined and that the study findings remained firmly grounded in the data.

### 2.3. Sampling Strategy

Participants were selected from private dental practitioners who took part in the study RESTO DATA DPBRN (15), an observational clinical study within the RECOL network conducted by 40 private practitioners, from different age groups practicing in both urban and rural regions across France. Our sampling strategy was based on two factors: the diversity of their perspectives and experiences (maximum variation sampling) and their availability (convenience sampling). This sampling strategy captured a broad range of viewpoints while ensuring that practical considerations were taken into account. A total of seven participants, representing a variety of professional backgrounds and experiences, were interviewed [Table 1]. This sample size achieved data saturation. Data saturation was considered to be reached when the last two interviews did not generate new themes or relevant information, in relation to the categories already identified through the ongoing analysis, as validated by the research team.

### 2.4. Data Management

All interviews were conducted by a single researcher (AMC), either in person or via telephone, between March 2022 and February 2023. One interview was completed in two parts due to an unforeseen emergency that required a brief interruption.

Data collection and analysis were iterative, with constant comparison used to identify emerging themes, refine the interview guide, and develop the coding framework.

All interviews were audio-recorded. Before implementation, a semi-structured interview guide [Table 2] was developed.

The questions were simple and open-ended, designed to avoid influencing the participants’ answers. The guide was not followed rigidly; instead, questions were asked in the flow of the conversation, allowing ample room for spontaneous expression.

The audio recordings were transcribed using word processing software and anonymised before analysis. The data files were securely stored on a password-protected hard drive to ensure the confidentiality and integrity of the information.

### 2.5. Data Analysis

This research follows a constructivist paradigm [25], which posits that reality is socially constructed and subjective, emphasizing the co-creation of knowledge through the interaction between the researcher and the participants. To perform our analysis, we followed the six-point recommendations of Braun and Clarke [25], and all the data were analyzed through a structured analytical process and validated within the research team.

Phase 1 involved familiarizing ourselves with the data through repeated readings and transcription. Phase 2 consisted of analysis by two researchers (VS and AMC) to increase reliability, alongside the keeping of a journal and note-taking during interviews. Items were coded starting from the most specific and then grouped together to code more broadly. Phase 3 involved generating themes inductively from the raw interview data. Phase 4 involved a team review of the themes, incorporating data triangulation to enhance trustworthiness. Regular discussions among the researchers facilitated the identification of common themes and the resolution of interpretative discrepancies. This process enriched the analysis while strengthening the validity and reliability of the research findings. Phase 5 involved defining and naming the themes, while phase 6 entailed writing up the results and citing verbatim data. The MaxQDA 2022 (VERBI Software, Berlin, Germany) was used to facilitate coding by breaking down the verbatim data into meaningful units.

## 3. Results

**Figure 1:**
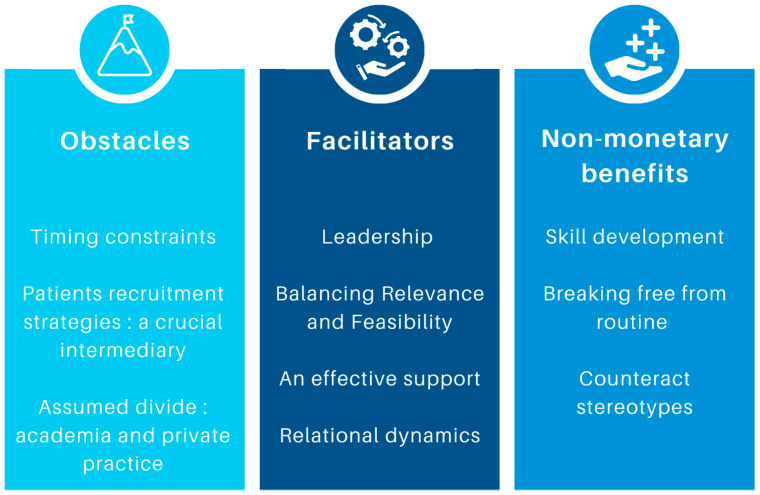
Key Themes Emerging from Private Dental Practitioners’ Experiences in Clinical Research.

Analysis of the interview data revealed several themes related to private dental practitioners’ experiences and perceptions of participating in the DPBRN. These themes and sub-themes were summarized in Figure 1.

### 3.1. Theme 1: Obstacles to Participating in a DPBRN

The analysis of the quotations reveals several obstacles that research studies conducted in private practices should overcome [Table 3].

#### 3.1.1. Timing Constraints

Despite a relatively good integration into the practice’s life, the RESTO DATA DPBRN study was not experienced in the same way by all respondents: time was actually a crucial factor in multiple ways. Some respondents indeed found that the research did not significantly impact their daily routine, as they already spent a lot of time with their patients during consultations. For these practitioners, participating in the research was a natural extension of their usual practice. However, others found that the research placed an additional burden on their already busy schedules. They had to adapt their consultation times and arrange to collect the necessary data, which was time-consuming and stressful. Moreover, the pressure of the schedule varies among the RESTO DATA DPBRN investigators. Some may have more flexibility in their schedules, while others may have more difficulties. This can affect their ability to prioritize and dedicate time to the study, potentially leading to variability in data collection and study implementation. Finally, practitioners felt that the timing of the research was sometimes not ideal for their patients. There may still be some hesitation or uncertainty among participants if a trusting relationship has not been established, indicating the importance of building trust before proposing to enter the study.

#### 3.1.2. Patients’ Recruitment Strategies: When the Practitioner Becomes the Crucial Intermediary

It is worth noting that the investigators involved in the RESTO DATA DPBRN study may have used different strategies to present the research study to potential participants. Some may have emphasized their own credentials or expertise, while others may have focused on the significance of the research question. Still, others may have downplayed the level of commitment required from patients, perhaps in an effort to make the study seem less daunting or time-consuming or insisted on the benefit for the patient to participate in the study. Finally, for some practitioners, presenting the study to patients was a source of stress and discomfort. This anxiety decreased over time as they became more familiar with the study protocol and recruitment process. Therefore, the quotes highlight the potential for miscommunication and discomfort in research recruitment, and suggest that practitioners’ own perceptions. They also suggest that practitioners’ own perceptions and attitudes can influence how they present the study to patients, which could affect the representativeness of the study sample.

#### 3.1.3. Assumed Divide: Academia and Private Practice

Some respondents reported a significant gap between academia and the private sector. They initially attributed research to academics, citing barriers such as the intensity and duration of studies, and institutional inertia. Despite the positive collaborative dynamic, respondents noted that the academic world turning towards the private world was *“sufficiently rare to make them want to help” (Participant G)*, indicating that they typically feel excluded from research matters. This sentiment was further reinforced by their belief that they could provide valuable data for research. They were confident that figures from ‘real-life practices’ could offer an alternative perspective to that of the hospital environment. This role of ‘data guardian’ was important to them. While the RESTO DATA study was seen as an opportunity to bridge this gap between private practices and the hospital setting, an underlying misunderstanding persisted.

### 3.2. Theme 2: Facilitators to Participating in a DPBRN

Participants identified several key factors that facilitated their participation in the RESTO DATA DPBRN study [Table 4]. These facilitators played an important role in encouraging their involvement and helped to create a positive research experience.

#### 3.2.1. Leadership and Charisma of Project Leaders

Most respondents emphasized the strong connection they had with the project leaders (BG and FD). They were able to communicate their passion and enthusiasm for research, which fostered greater involvement and commitment. Their charisma enabled them to rally people around them and create a sense of cohesion within the research team.

#### 3.2.2. Enthusiastic Engagement in Knowledge Production

Participants highlighted *‘the intelligence of having done something very serious and qualitative but not too constraining so that it could be incorporated into the activity of “dental office”’ (Participant F)*. The investigators of the RESTO DATA DPBRN study have demonstrated a commitment to conducting research in accordance with ethical and scientific principles, which has likely strengthened their dedication to the DPBRN. It also emphasizes the value of creating research opportunities that align with the needs and interests of private practice dentists. They found meaning in their participation. For some participants, this study likely successfully balanced rigour and quality with feasibility and practicality, making it easier for dentists to incorporate research into their practice.

#### 3.2.3. Effective Logistical Assistance

Respondents unanimously agreed on the importance of support throughout the study: a group messaging platform connecting the study’s investigators and the research team was widely acclaimed. This support enabled the investigators to acquire the necessary skills to obtain quality data and provided them with the emotional support needed to prevent dropouts.

#### 3.2.4. Relational Dynamics

In the context of their participation in the research, many participants highlighted the importance of relationships as a key factor. This highlights the significant impact of human interactions on the research experience and the way individuals engage in the process.

Firstly, this study focuses on the network of relationships between researchers who have known each other for a long time and share friendships. Furthermore, building relationships with the research team fosters trust, encouraging participants to fully engage with the research. The RECOL network seems to provide the necessary space. Respondents emphasized the dynamics of exchange, describing research that brings people together, thus reinforcing the sense of solidarity and collaboration within the RECOL community.

### 3.3. Theme 3: Personal Gains from DPBRN Participation

The study RESTO DATA DPBRN did not provide compensation to investigators for their participation. Although paying dentists could be an effective motivator, the testimonies collected during interviews emphasize the various personal benefits that research participation can offer participants, beyond any financial compensation [Table 5].

#### 3.3.1. Skill Development

Participants reported that taking part in the study had provided them with valuable opportunities for personal and professional development. They noted that the research experience allowed them to improve clinical skills, which in turn increased their confidence and competence in their daily practice. For instance, some participants reported that taking part in the study made them pay closer attention to certain aspects of their intraoral examinations. One participant noted *‘I realized that I was looking for a lot less’ (Participant C)* when discussing how he checked the quality of dental restorations in their patients’ mouths.

#### 3.3.2. Breaking Free from Routine

Participants reported that taking part in this study gave them valuable opportunities to socialize and network. They noted that their research experience had allowed them to connect with people outside of their usual social and professional circles. Some participants also expressed that taking part in research allowed them to challenge themselves and broaden their perspectives by doing things outside their usual routine, *‘working the brain a bit for things other than everyday stuff’ (Participant G)*.

#### 3.3.3. Counteract Stereotypes

One subtheme that emerged is the challenge of stereotypes in the dental profession. Participants highlighted the potential for research participation to counteract negative stereotypes about their profession and to demonstrate their commitment to advancing knowledge. They are no longer viewed as lone practitioners, but as members of a wider network of healthcare professionals who share the same goal: to improve health and contribute to society. They may even feel personally invested in the outcomes of the research and be surprised or taken aback by unexpected findings. While some participants were not surprised by the results, others expressed disappointment at the prevalence of dental caries, revealing an underlying belief that younger generations would be free from this issue. It emphasizes the transformative potential of research by deconstructing representations from the perspectives of both patients and investigators.

## 4. Discussion

This qualitative study revealed three themes relating to the experiences and perceptions of private dental practitioners involved in clinical research: (1) the obstacles faced in private practice research, (2) the key elements that facilitated private practitioners’ participation, and (3) the personal benefits of participation beyond financial compensation.

It is clear that recruiting practitioners is a persistent challenge for DPBRNs. Without them, no research project can succeed [26,27]. Our findings are consistent with previous literature which highlights time constraints, logistical challenges, and a perceived lack of relevance as key obstacles [28,29]. In particular, Demarco et al. pointed out that clinical workload and concerns about disrupting routine practice are significant barriers to participation in dental research [28]. Additionally, Mungia et al. emphasized the importance of strategic efforts such as sustained investment in infrastructure, effective engagement strategies, and bespoke organizational skills to ensure the productive operation of PBRNs [30]. This aligns with our study, in which participants emphasized the importance of the social nature and relational dynamics of a PBRN.

Our study identified several factors that facilitate the participation of private practitioners, including effective communication, research relevance, and non-judgmental relationships between practitioners and the research team. These findings resonate with those of Hopper et al., who noted that a supportive research environment and clear communication with academic researchers are essential for sustained engagement [31]. In addition, Mungia et al. highlighted the importance of designing studies that are practical and relevant to everyday clinical practice, which increases the likelihood of participation [30].

The testimonies collected in our study emphasized the various personal and professional benefits of participating in PBRNs, which extend beyond financial compensation. Participants reported improvements in their clinical skills, increased confidence and a sense of professional growth. These findings are consistent with those of Hopper et al. [31], who noted that dentists appreciated the opportunity to refine their clinical procedures and stay updated with current research. Our results also highlighted the role of research participation in reducing professional isolation and broadening practitioners’ perspectives. This aligns with Demarco et al., who emphasized that participation in research can break the monotony of daily practice and foster a sense of community among practitioners [28]. Furthermore, Mungia et al. pointed out that the ability to contribute to the evidence base and improve patient outcomes is a powerful motivator for clinicians.

Interestingly, while financial compensation or credits for continuum education are often cited as a motivator for research participation [28,29], which was not a significant concern for our participants. This may be attributed to the study design, which was not overly time-consuming, and to the collaborative and supportive environment fostered by the research team. Our findings suggest that non-monetary incentives, such as skill development, professional recognition and networking opportunities, can be equally, if not more, effective in motivating participation.

One limitation of this qualitative study was the difficulty in data collection, particularly regarding the conditions and time allocated for interviews, which may have impacted the richness and depth of the data obtained. Additionally, the sample size of seven participants, while sufficient for achieving data saturation, may limit the generalizability of the findings. Moreover, some researchers were involved in the network under study; although reflexive practices and team discussions were used to enhance rigour, some influence on interpretation cannot be entirely excluded.

More importantly, our findings are specific to participation in a DPBRN observational study (RESTO DATA study). Perceptions may differ for more complex study designs, such as randomized controlled trials or longitudinal studies, which require greater time commitments, ethical considerations, and potential restrictions on clinical autonomy. Lastly, perceptions of research participation may differ across medical specialties due to differences in clinical practice and organization of care, although certain insights may be transferable to other clinical contexts. Further research is needed to explore these contexts and provide a more comprehensive understanding of the factors that influence participation in DPBRNs.

Based on our findings and the existing literature, we propose several practical recommendations to enhance the effectiveness and sustainability of future DPBRNs and their related studies. First, it is crucial to promote a culture of research from initial training onward. This approach can improve clinical care and encourage practitioners to participate in research studies [32]. Early exposure to research concepts and methods is likely to increase engagement and foster a lifelong commitment to evidence-based practice. One effective way to make research more accessible and relatable is to develop educational videos and case studies featuring healthcare professionals sharing their research experiences.

It also seems essential to provide supportive resources and tools that simplify the research process. Tools that facilitate patient recruitment, data collection, and workflow integration can reduce the burden on practitioners and encourage participation [29].

Personalized training and mentorship programs are essential for developing the research skills and confidence of practitioners. Providing hands-on workshops, peer mentoring, and access to research experts can help bridge the gap between clinical work and research activities [30]. To further motivate participation, offering incentives such as continuing education credits, professional recognition, or financial compensation is likely to be effective. Creating networking opportunities between private practitioners and academic researchers helps reduce professional isolation and fosters a sense of community [7]. In medical PBRNs, regular meetings, conferences, and collaborative platforms have strengthened these connections and improved research outcomes [7]. Improving the involvement of dental surgeons in PRBNs is crucial because, as key public health actors, oral health professionals also help reduce inequalities in access to care and support the inclusion of oral health in broader health policy frameworks [33]. Finally, it is important to involve practitioners in co-constructing research by identifying relevant research questions and assessing the feasibility of study protocols [21].

## 5. Conclusions

This study provides important insights into the factors influencing practitioners’ willingness to engage in clinical research. Key themes include the barriers posed by time constraints, administrative burdens, and the need for a supportive network and resources. Dental Practice-Based Research Networks can better support practitioners by addressing these challenges through tailored strategies such as accessible training, practical incentives, and opportunities for collaboration. Moreover, the present findings may be generalized across a wide range of healthcare disciplines. By demonstrating consistent trends relevant to clinical research, this study provides researchers, regardless of their area of specialization, with guidance that may inform the implications of investigators.

## Figures and Tables

**Table 1:** Characteristics of participating dentists.

Participant ID	Age	Gender	Years of Experience	Practice Location	Involvement of the Dental Assistant	Previous Research Experience	Specialization
**A**	30–35	Male	10	Urban	Yes	Yes	General dentistry (with an implantology orientation)
**B**	35–40	Female	15	Suburban	Yes	No	General dentistry
**C**	30–35	Female	10	Urban	Yes	No	General dentistry
**D**	45–49	Female	20	Rural	Yes	No	Endodontics
**E**	50–55	Female	25	Suburban	No	Yes	General dentistry
**F**	40–45	Male	15	Rural	No	Yes	General dentistry
**G**	45–49	Female	20	Suburban	Yes	Yes	General dentistry

**Table 2:** Interview guide.

Items	Questions
Introduction and motivation	▪ What were your expectations going into the study? ▪ How did you first hear about the study, and what information were you given before deciding to participate?
Experience	▪ Can you tell me about your experience? ▪ Were there any challenges or barriers you faced while participating in the study? ▪ Can you tell me about your experience communicating with the research team and asking questions throughout the study?
Impact on professional life	▪ How did participating in the study impact your daily life at the dental practice? ▪ How did your participation in the study affect your working relationship with your dental assistant, if at all? ▪ Did your dental assistant express any concerns or reservations about your participation in the study?
Impact on the patient relationship	▪ Did you find that participating in the study changed the way you communicated with your patients? ▪ How did patients react when they learned that you were participating in a research study? ▪ Did you feel that your involvement in the study had any impact on the rapport you had established with your patients?
Collaboration and exchange with peers	▪ How did you feel about collaborating with other dentists who were also involved in the study? ▪ How did the opportunity to share experiences and insights with other dentists involved impact your experience?
Tools and results	▪ Can you describe how you used the technical support tools provided for the study? Were there any challenges or benefits to using these tools? ▪ Can you tell me about your thoughts and feelings regarding the results of the research study?
Futures participations	▪ Is there anything else you would like to share about your experience? ▪ Why or why not would you consider participating in another dental research study in the future? ▪ Why or why not would you recommend participation in a dental research study to a colleague? ▪ Are there any other thoughts or perspectives that we have not discussed yet?

**Table 3:** Obstacles to participating in a DPBRN.

Sub-Themes	Quotations
Timing constraints	▪ *I would spend 1 h each time, so it’s true that we had to adjust the consultation times for the initial consultations in advance, knowing that we have almost 4 months of delay at the office so the appointments were already set, so it was a bit painful to set. (B)* ▪ *In a private practice setting, it’s extremely burdensome.* ▪ *My practice was completely suited for this. (A)* ▪ *It hasn’t really changed my daily routine because I have a very particular practice where I already spent a lot of time with my patients during consultations. (A)* ▪ *In the agenda, we had to plan, but it wasn’t awful (B)*
Criticality of study presentation to patients	▪ *It’s the way you present it (…) I had my academic status, so that always carries a bit of weight. (E)* ▪ *When you really explain to them that it’s something we need, that it’s important. (E)* ▪ *Just a visual study that doesn’t involve any clinical procedure. (E)* ▪ *It was still easier at the end (…) more in terms of presenting it, it was less stressful to talk about it. (B)* ▪ *Then I would pitch it as an extremely thorough examination of their mouth and (…) who would refuse to have a thorough examination of their mouth? (A)*
Assumed divide: academia and private practice	▪ *I also didn’t want to do very long, academic research projects for which I didn’t have the stature (but) it opened a door. (A)* ▪ *We can participate in research without it being unpleasant and extremely burdensome (F)* ▪ *This type of research is generally done in a hospital setting, so the dental health status of the French population is based on very specific patients who are followed at the hospital, and it’s not at all representative of the real population—(…) this study will help to readjust things a bit, to understand the true state of the French population (G)*

**Table 4:** Facilitators to participating in a DPBRN.

Sub-Themes	Quotations
Leadership and charisma of project leaders	▪ *It’s inevitably due to the drive that B. and F. can provide, along with their notoriety. (E)* ▪ *When B. proposes something to you, I support her to the maximum. (F)* ▪ *I didn’t feel like abandoning them halfway, even though honestly it required a significant effort from me. (B)* ▪ *It’s necessary to get people on board, which isn’t easy. (E)*
Enthusiastic engagement in knowledge production	▪ *The intelligence of having created something very serious and high-quality but not too constraining, so that it could be incorporated into the activity of a private practice. (F)* ▪ *Here, we don’t have to deal with anything; we arrived, put our feet under the table, so to speak, and we’re moving forward with the data. So all the aspects related to architecture, eCRF, and all the mandatory declarations… we didn’t have to worry about any of that. (F)* ▪ *It was always a question I had asked myself and could finally get an answer to. (E)*
Effective logistical assistance	▪ *We would immediately get a response, meaning that we would send a message and always have someone call us back the next day to resolve the problem. (G)* ▪ *To say where we were at, how we were progressing, the difficulties we were encountering. (G)* ▪ *I think that if we had struggles with the first ones and it didn’t improve afterwards, and we were left struggling, I think we would have given up very quickly. (G)*
Relational dynamics	▪ *For me, the relational aspect is crucial for getting people to adhere to this kind of project. (E)* ▪ *The existing network of friendships played a significant role. (F)* ▪ *It brought… motivation, a sense of group enthusiasm. (A)*

**Table 5:** Personal gains from DPBRN participation.

Sub-Themes	Quotations
Skill development	▪ *I find it interesting from a scientific point of view to participate in this. (B)* ▪ *‘I’ve been practicing for 10 years and I realized that I was looking a lot less, it forced me to be more attentive. (C)*
Breaking free from routine	▪ *There are people to meet, and I think that’s important too, we’re not just going to work with people we already know. (E)* ▪ *I think it’s an excellent initiative that helps fill a void. (F)* ▪ *I’m interested in doing some things, working the brain a bit for things other than everyday stuff, you know, opening up the mind a bit. (G)*
Counteract stereotypes	▪ *We have a bit of a reputation for wanting to make money in private practice, whereas in reality we are quite open-minded. (C)* ▪ *Patients were quite pleased to see a practitioner who is involved in research. (A)* ▪ *Conducting this assessment at the beginning of the 21st century was very important. (E)* ▪ *I am disappointed, we are far from the Scandinavian countries. (E)* ▪ *I was convinced that the new generations would not have any carious lesions. (E)*

## Data Availability

The original contributions presented in this study are included in the article/Supplementary Material. Further inquiries can be directed to the corresponding author.

## Supplementary Materials

The following supporting information can be downloaded at: https://www.mdpi.com/article/10.3390/healthcare14080979/s1, File S1: Standards for reporting qualitative research (SRQR).
