# Supplementary material for: Private Dental Practitioners’ Experience in a Dental Practice-Based Research Network: A Qualitative Evaluation
Source: Healthcare (Basel). 2026 Apr 8;14(8):979. doi: 10.3390/healthcare14080979 (PMC13115664; doi:10.3390/healthcare14080979)
Supplement: Supplementary file 1 [file healthcare-14-00979-s001.zip › healthcare-4111576-supplementary.pdf]

Standards for Reporting Qualitative Research (SRQR)

S1 – Title
Private dental practitioners' experience in a Dental Practice-Based Research Network: A qualitative evaluation.

S2 – Abstract / Summary
Qualitative study exploring motivations and satisfaction of investigators involved in the RESTO-DATA study within the RECOL practice-based research network in France. Data were collected through semi-structured interviews and a focus group to explore experiential and psychological factors influencing participation in clinical research.

S3 – Background and rationale
Dental clinical research in France is mainly hospital-based, with limited involvement of private practitioners. Practice-Based Research Networks (PBRNs) may generate real-world evidence, but motivations and barriers to practitioner participation remain insufficiently documented.

S4 – Objectives
To identify factors influencing dentists' participation in clinical research. Secondary objectives were to explore barriers and facilitators to future participation and to identify investigators' expectations.

S5 – Qualitative approach and research design
This study followed a qualitative research design using semi-structured interviews and thematic analysis to explore dentists' perceptions of participation in a DPBRN study.

S6 – Researcher characteristics and reflexivity
Both lead investigators (AMC and VS) are dentists with more than 10 years of experience in a hospital-university setting. Additionally, one investigator (AMC) participated as a practitioner-researcher in the clinical study from which the sample for this research was drawn, while the other (VS) serves on the association's board. Throughout all stages of the research, they (AMC and VS) engaged in continuous self-reflection and discussions with the research team (other co-authors) to ensure that their perspectives and assumptions were critically examined and that the study findings remained firmly grounded in the data.

S7 – Context
The study was conducted within the RESTO-DATA multicenter observational study implemented through the RECOL dental practice-based research network.

S8 – Sampling strategy
Purposive and convenience sampling among RESTO-DATA investigators. Seven interviews were conducted until data saturation.

S9 – Ethical considerations
Oral and write informed consent was obtained from participants. Interviews were audio-recorded with permission and anonymized.

S10 – Data collection methods
Semi-structured interviews (~30 minutes) conducted by the same researcher, either face-to-face or by phone. Data collection started in March 2022.

S11 – Data collection instruments
An interview guide was used. Interviews were audio-recorded and transcribed for analysis.

S12 – Participants
Participants were selected from among private dental practitioners who took part in the study RESTO DATA DPBRN (15), an observational clinical study within the RECOL network conducted by 40 private practitioners, from different age groups practicing in both urban and rural regions across France. Our sampling strategy was based on two factors: the diversity of their perspectives and experiences (maximum variation sampling), and their availability (convenience sampling). This sampling strategy captured a broad range of viewpoints while ensuring that practical considerations were taken into account. No refusals were reported.

S13 – Data processing
Interviews were transcribed verbatim and anonymized prior to analysis.

S14 – Data analysis
Inductive thematic analysis following Braun and Clarke's six-phase framework. Two researchers independently coded transcripts using MaxQDA software.

S15 – Techniques to enhance trustworthiness
Reliability ensured through dual coding, triangulation of interviews and adherence to SRQR checklist.
